# Effect of a novel vital sign device on maternal mortality and morbidity in low-resource settings: a pragmatic, stepped-wedge, cluster-randomised controlled trial

**DOI:** 10.1016/S2214-109X(18)30526-6

**Published:** 2019-02-14

**Authors:** Nicola Vousden, Elodie Lawley, Hannah L Nathan, Paul T Seed, Muchabayiwa Francis Gidiri, Shivaprasad Goudar, Jane Sandall, Lucy C Chappell, Andrew H Shennan, CRADLE Trial Collaborative Group, Monice Kachinjika, Doreen Bukani, Jane Makwakwa, Grace Makonyola, Adrian Brown, Paul Toussaint, Adeline Vixama, Grace Greene, Carwyn Hill, Emily Nakiriija, Doreen Birungi, Noela Kalyowa, Dorothy Namakula, Josaphat Byamugisha, Annettee Nakimuli, Nathan Mackayi Odeke, James Ditai, Julius Wandabwa, Fatmata Momodou, Margaret Sesay, Patricia Sandi, Jeneba Conteh, Jesse Kamara, Matthew Clarke, Rebecca Best, Josephine Miti, Mercy Kopeka, Bellington Vwalika, Martina Chima, Thokozile Musonda, Christine Jere, Sebastian Chinkoyo, Violet Mambo, Yonas Guchale, Lomi Yadeta, Feiruz Surur, Geetanjali M Mungarwadi, Sphoorthi S Mastiholi, Chandrappa C Karadiguddi, Umesh Charantimath, Mrutyunjaya Bellad, Natasha Hezelgrave, Kate E Duhig

**Affiliations:** a Department of Women and Children's Health, School of Life Course Sciences, Faculty of Life Sciences and Medicine, King's College London, London, UK; b Department of Obstetrics and Gynaecology, College of Health Sciences, University of Zimbabwe, Harare, Zimbabwe; c Women's and Children's Health Research Unit, KLE Academy of Higher Education and Research, Jawaharlal Nehru Medical College, Belgaum, Karnataka, India

## Abstract

**Background:**

In 2015, an estimated 303 000 women died in pregnancy and childbirth. Obstetric haemorrhage, sepsis, and hypertensive disorders of pregnancy account for more than 50% of maternal deaths worldwide. There are effective treatments for these pregnancy complications, but they require early detection by measurement of vital signs and timely administration to save lives. The primary aim of this trial was to determine whether implementation of the CRADLE Vital Sign Alert and an education package into community and facility maternity care in low-resource settings could reduce a composite of all-cause maternal mortality or major morbidity (eclampsia and hysterectomy).

**Methods:**

We did a pragmatic, stepped-wedge, cluster-randomised controlled trial in ten clusters across Africa, India, and Haiti, introducing the device into routine maternity care. Each cluster contained at least one secondary or tertiary hospital and their main referral facilities. Clusters crossed over from existing routine care to the CRADLE intervention in one of nine steps at 2-monthly intervals, with CRADLE devices replacing existing equipment at the randomly allocated timepoint. A computer-generated randomly allocated sequence determined the order in which the clusters received the intervention. Because of the nature of the intervention, this trial was not masked. Data were gathered monthly, with 20 time periods of 1 month. The primary composite outcome was at least one of eclampsia, emergency hysterectomy, and maternal death. This study is registered with the ISRCTN registry, number ISRCTN41244132.

**Findings:**

Between April 1, 2016, and Nov 30, 2017, among 536 223 deliveries, the primary outcome occurred in 4067 women, with 998 maternal deaths, 2692 eclampsia cases, and 681 hysterectomies. There was an 8% decrease in the primary outcome from 79·4 per 10 000 deliveries pre-intervention to 72·8 per 10 000 deliveries post-intervention (odds ratio [OR] 0·92, 95% CI 0·86–0·97; p=0·0056). After planned adjustments for variation in event rates between and within clusters over time, the unexpected degree of variability meant we were unable to judge the benefit or harms of the intervention (OR 1·22, 95% CI 0·73–2·06; p=0·45).

**Interpretation:**

There was an absolute 8% reduction in primary outcome during the trial, with no change in resources or staffing, but this reduction could not be directly attributed to the intervention due to variability. We encountered unanticipated methodological challenges with this trial design, which can provide valuable learning for future research and inform the trial design of future international stepped-wedge trials.

**Funding:**

Newton Fund Global Research Programme: UK Medical Research Council; Department of Biotechnology, Ministry of Science & Technology, Government of India; and UK Department of International Development.

## Introduction

Maternal mortality remains a challenge worldwide, especially in low-resource settings, where in 2015, an estimated 303 000 women died from complications of pregnancy and childbirth.^1^ The leading direct causes of maternal mortality are obstetric haemorrhage (27·1%), hypertensive disorders (14·0%), sepsis (10·7%), and abortion complications (7·9%).^2^ Simple, evidence-based interventions are available for the majority of these conditions.^3^ However, in low-resource settings, delays in presenting to care, reaching care, and receiving this care all contribute to high maternal mortality.^4^

Vital sign measurement is the first step in recognising women at risk of deterioration (particularly from hypertension, obstetric haemorrhage, and sepsis) and, therefore, in initiating treatments that can prevent potentially catastrophic maternal and perinatal complications.^5^ Early warning systems allow for tracking of vital signs to alert health-care providers to abnormalities and allow earlier action. They are widely used in high-income settings.^6^ Several studies have found early warning systems to be beneficial in predicting maternal morbidity and mortality, but these studies are generally small and retrospective.^7^, ^8^, ^9^, ^10^, ^11^, ^12^, ^13^ Only one prospective, non-randomised study^14^ has shown that implementation of a paper-based early warning system across six pilot hospitals resulted in a significant reduction in a composite of maternal morbidity compared with standard care at non-pilot sites in the USA. However, to be effective, early warning systems require accurate measurement and documentation of vital signs followed by calculation of risk and appropriate escalation or action. In low-resource settings, inadequate access to reliable equipment,^15^, ^16^ overstretched staff,^17^ and poor understanding of pregnancy complications and monitoring of vital signs^18^ can potentially lead to delay in identifying and initiating treatment in women most at risk.

**Research in context**

**Evidence before this study**

We searched PubMed for original articles published in English before July 1, 2018, with the search terms “maternal OR maternity OR pregnancy AND early warning”. We identified eight studies (seven undertaken in high-resource settings), which examine the predictive capacity of early warning systems in pregnancy. No clinical trials or systematic reviews were identified. However, it is widely acknowledged that delays in recognising and initiating treatment for pregnancy complications contribute to maternal mortality. Therefore, early warning systems are consistently recommended in high-resource settings. In low-resource settings, inadequate access to accurate vital signs equipment and trained staff further adds to delays in detecting pregnancy complications. To our knowledge, no study has yet investigated novel solutions that integrate early warning systems into accurate equipment to reduce maternal morbidity and mortality in low-resource settings.

**Added value of this study**

This pragmatic, stepped-wedge, cluster-randomised controlled trial provides the first assessment of a novel early warning system and accurate vital signs device in preventing maternal morbidity and mortality in low-resource settings. In ten clusters in eight low-income and middle-income countries, introduction of the device with an educational package into routine maternity care reduced a composite of maternal morbidity and mortality, but the variation in event rates between and within clusters over time meant this reduction could not be attributed to the intervention, despite more than 4000 primary outcome events (maternal death, eclampsia, or hysterectomy) among a population of more than half a million pregnant women. Very few trials of a similar size have been done in maternity populations and, to our knowledge, none has investigated an early warning system.

**Implications of all the available evidence**

The existing evidence has shown that early warning systems might be beneficial in detecting pregnancy complications earlier in high-resource settings. This trial has shown a reduction in maternal morbidity and mortality during the trial period, with no change in resources or staffing. However, the unexpected degree of variability within clusters over time and between clusters meant that this reduction cannot be attributed to the intervention. Evaluation of the intervention in individual countries might elucidate mechanisms by which it affects outcomes. To our knowledge, this is the first stepped-wedge cluster randomised controlled trial across multiple countries and continents; the design was chosen after careful consideration, including the intention to leave all ten sites equipped with vital sign monitoring devices. We encountered unanticipated methodological challenges, which provide valuable learning for future research and inform the trial design of future international stepped-wedge trials.

**Figure 1:**
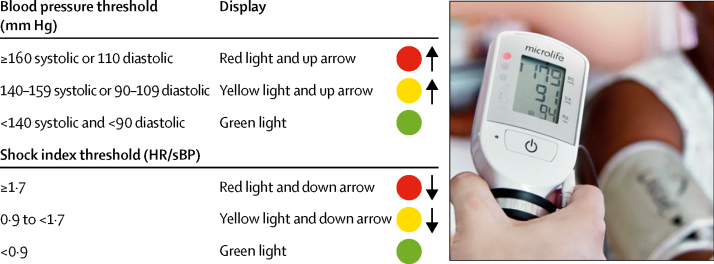
Thresholds that trigger the traffic light early warning system on the CRADLE Vital Sign Alert HR=heart rate. sBP=systolic blood pressure.

The CRADLE Vital Sign Alert (VSA) is a semi-automated device that measures blood pressure and heart rate, and calculates shock index (heart rate divided by systolic blood pressure). This device has been extensively tested and is validated as accurate in pregnant women according to international protocols, including in women with high and low blood pressure.^19^, ^20^, ^21^, ^22^ Qualitative implementation evaluation in low-resource settings has determined that it is easy to use, robust, and suitable for use by any cadre of health-care provider, even those without extensive training, such as community health workers.^23^ Results are displayed digitally and on a traffic light early warning system, which indicates abnormal vital signs (figure 1).^23^ This ease of use is important in low-resource settings, where routine clinical tasks, such as vital signs measurement, are often undertaken by health-care workers, students, or volunteers with minimal training, and where community health workers also play a vital part in maternity care, often being the first point of contact and an essential link to clinical services.^3^, ^24^

The primary aim of the trial was to determine whether implementation of the CRADLE VSA and an education package into community and facility maternity care in low-resource settings could reduce a composite of all-cause maternal mortality or major morbidity (eclampsia and hysterectomy).^25^ We hypothesised that this intervention would improve the quality of care by increasing the number of trained health-care providers (including those who informally support routine vital signs measurement, such as volunteers) and by providing reliable equipment, thus increasing the number of women who receive vital signs monitoring and subsequent management of pregnancy complications. This trial was preceded by a 6-month feasibility study to develop and improve the intervention and implementation strategies,^26^ informed by guidance from the UK Medical Research Council for evaluation of complex interventions.^27^

## Methods

### Study design

This pragmatic, stepped-wedge, cluster-randomised controlled trial evaluated the CRADLE VSA intervention in routine maternity care in low-resource settings. All methods were predefined and published,^25^ with no important methodological changes. Clusters were purposely selected to represent a range of low-resource settings. Ten clusters across eight countries were identified and agreed to participate: Addis Ababa in Ethiopia, Cap Haitien in Haiti, Freetown in Sierra Leone, Harare in Zimbabwe, Gokak in India, Kampala and Mbale in Uganda, Lusaka and Ndola in Zambia, and Zomba plus the Southern Region in Malawi. Each cluster comprised at least one urban or peri-urban secondary or tertiary health facility that provides comprehensive emergency obstetric care with multiple peripheral facilities that refer to the central hospital.^25^ Facilities were identified by the local primary investigators as the main facilities that refer to the central hospital within a feasible geographical area. Community health-care providers were included in implementation in clusters where they were supported at a district level and were active in routine maternity care provision (Ndola and Cap Haitien). Clusters crossed over from control to the CRADLE intervention in one of nine steps at 2-monthly intervals, with CRADLE devices replacing existing equipment at the randomly allocated timepoint. Ethics approval was granted by the King's College London (UK) Research Ethics Subcommittee (LRS-14/15-1484) and in all countries before the start of the trial (appendix). Institutional-level consent on behalf of the cluster was obtained.^28^ See reference 25 for the protocol.

### Participants

All health-care providers working in the cluster facilities had access to the intervention. All women identified as pregnant or within 42 days of delivery, presenting for maternity care in a cluster facility or to community health-care providers, were exposed to the intervention. There were no exclusion criteria.

### Randomisation and masking

The unit of randomisation was the cluster. A restricted method of randomisation was used such that there was zero rank correlation between events per month and order of randomisation to minimise imbalance between intervention and pre-intervention periods due to anticipated variation in the primary event rate between clusters. A computer-generated randomly allocated sequence run by the CRADLE statistician (PTS) determined the order in which the clusters received the intervention. All clusters were masked to the order until 2 months before receiving the intervention, when the next cluster to receive the intervention was informed. Because of the nature of the intervention, this trial was not masked. The two smallest clusters were randomised at the same time. Data were gathered monthly, with 20 time periods of 1 month.

### Procedures

Before the intervention, various medical devices (where previously available) were used in routine maternity care, with management by local guidelines (pre-intervention period). The CRADLE VSA and training package was iteratively developed and piloted as previously described.^25^ At the randomly allocated timepoint, the training package was delivered in interactive group sessions to health-care providers from each of the clinical areas in the cluster facilities. Some health-care providers in each cluster became CRADLE Champions and provided ongoing training and support in their clinical areas. The local implementation team provided regular support to all facilities, with at least monthly contact. Existing equipment for measuring vital signs was usually removed from clinical use unless specific functions were needed (eg, repeated automated measures in a high-dependency unit). We did not include a transition period; outcomes occurring after implementation start were allocated to the intervention group (post-intervention period). After the end of the trial, clusters were able to continue using the intervention.

Each cluster included primary (first point of access), secondary (first referral point), and tertiary facilities (specialty referral facility). Maternity unit staffing levels (doctors, nurses, midwives, clinical officers, and community health-care providers in Ndola and Cap Haitien, where active in routine care) and availability of key resources (magnesium sulphate, intensive care unit beds, and capacity for blood transfusion) were recorded throughout the trial period. Major changes to infrastructure, patient payment requirements, or environmental conditions were systematically evaluated each month in each site. Service impact was assessed by the proportion of women referred from peripheral facilities to higher-level care (collected for a 4-week period before and 3 months after implementation).

Before the start of the trial, data collection methods were optimised based on existing resources available in each site. Outcomes were triangulated across multiple sources (including referral registers, ward registers, patient records, local mortality and morbidity records, and active case finding) to ensure data completeness. Source data consistency and quality were monitored by the local research team, with a proportion verified by the UK research team. There were no formal stopping rules. The trial ended after 20 months as planned.

### Outcomes

The primary outcome was a rate of a composite of maternal mortality or major morbidity: at least one of maternal death (all-cause mortality), eclampsia (occurrence of generalised convulsions with increased blood pressure in the absence of epilepsy or another condition predisposing to convulsions), or emergency hysterectomy (surgical removal of all or part of the uterus) per 10 000 deliveries per month, occurring during pregnancy, labour, or within 42 days of delivery. Predefined secondary maternal outcomes were the individual components of the primary outcome (eclampsia, emergency hysterectomy, and maternal death), intensive care unit admission (defined as admission or referral to a specific intensive care unit or equivalent defined highest-level care environment), and stroke. Secondary perinatal outcomes, collected per 1000 women with a primary outcome, were the rate of stillbirths and neonatal deaths. Neonatal death was defined as death of a live born infant within 28 days of delivery at or after 28 weeks of gestation. Stillbirth was defined as born without signs of life at or after 28 weeks of gestation. Because the intervention itself was not considered likely to lead to adverse events, and all major adverse pregnancy complications were included as outcomes, no additional adverse event reporting was undertaken. There were no changes to prespecified outcomes during recruitment, and only prespecified analyses were undertaken.

### Statistical analysis

Sample size estimation was informed by data from the feasibility phase and carried out using Hemming and Girling's methods.^29^ Assuming 4000 deliveries per cluster per month, with nine clusters, each observed for 20 months, and a baseline event rate of 1% with a reduction to 0·75% post-intervention, 2450 outcome events were required to have power of 95% (selected to account for cluster variability in a stepped-wedge randomised controlled trial). A coefficient of variation of 0·4 (judged to be high, but plausible, based on our pilot data) and an intracluster correlation coefficient calculated as 0·0085 were selected for this study design.

We did the planned comparison using risk ratios, but it did not converge for the majority of results. This finding is common in analyses of rates; therefore, results are reported as odds ratios (ORs) with 95% CIs. We used logistic regression with generalised estimating equations and a population-averaged model for the main analysis.^30^ For the primary analysis of the primary outcome and its individual components,^31^ we adjusted for three predictors: cluster (categorical), time from start of study (continuous; with an interaction between cluster and time so that each cluster had its own underlying time trend), and total time on the randomised intervention, with time before intervention given as zero (continuous). The analysis resulted in separate linear time trends in each cluster, as prespecified. The model for intervention analysis allowed for separate linear trends in each cluster before and after the intervention (or a change in trend at the time the intervention was introduced, known as linear splines or bent stick). This model was an amendment from the planned single linear trend in each cluster with a sudden change (or step) at the time of the intervention (no change in trend or slope, known as trend and step). We selected this alternative model because the unexpected variation led to greater instability in the analysis. The bent stick model achieved greater stability. Both analyses are presented and were adjusted for the same predictors. A significance level of 0·05 was used for all analyses.

As was prespecified in the protocol, we also analysed the results for individual clusters using fixed linear trend and a step at the start of intervention because bent stick models were not reliable for clusters that implemented the intervention early or late and therefore had few data points with or without the intervention. Because individual patient data were only collected for women with a primary outcome, we had to treat all other women as having no event and therefore had no information from which to estimate the extent of missing data. The possible number of women who might have had a primary outcome without being recorded was not expected to have changed following the intervention.

Sensitivity analysis removing data collected the week before and after implementation was originally intended to account for the learning phase following introduction of the intervention. However, this analysis proved impossible because only monthly delivery data could be collected. Analysis removing data collected at time periods during which there were major external influences was done as planned. Autoregressive correlation allows for decreasing correlations between observations over greater time periods; we did further analysis for alternative correlation structures as planned (appendix). We planned to adjust for any significant differences in the characteristics of clusters (number of facilities, obstetric resources, and personnel) before and after the intervention, but none were found. We calculated CIs using generalised estimating equations and robust standard errors adjusted for clustering. Statistical analyses were done in Stata, version 14.2 (by PTS). This study is registered with the ISRCTN registry, number ISRCTN41244132.

### Role of the funding source

The funders of the study had no role in study design, data collection, data analysis, data interpretation, or writing of the report. The corresponding author had full access to all the data in the study and had final responsibility for the decision to submit for publication.

## Results

**Figure 2:**
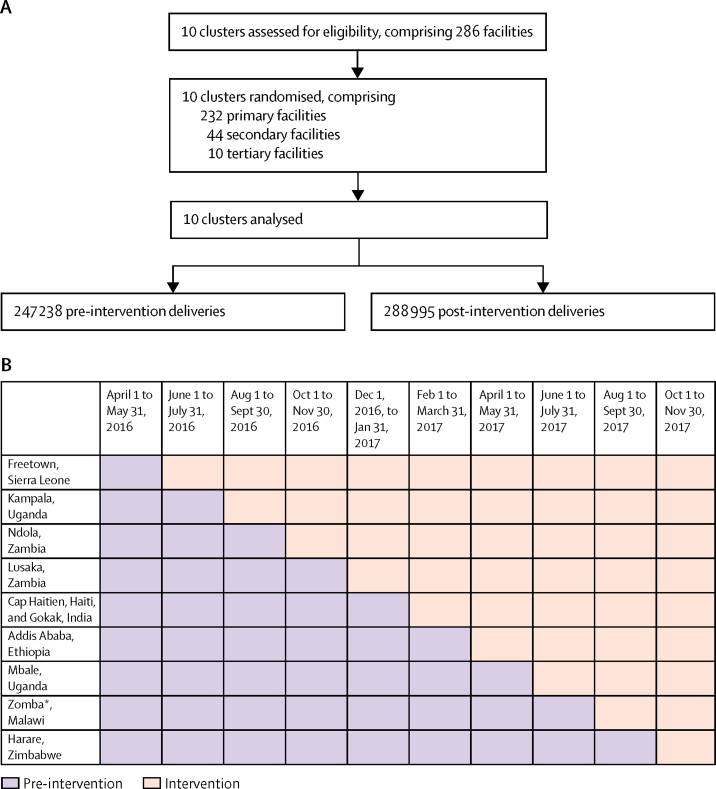
Trial profile (A) and randomisation schedule (B) Data were collected at each randomly allocated timepoint, irrespective of whether the intervention had been initiated. Eight primary and eight secondary facilities stopped providing maternity services or closed between the start of the trial and analysis. *Plus the Southern Region.

We approached and included ten clusters, which comprised 286 facilities (figure 2). Clusters were well balanced in both groups, with no significant differences in their characteristics, including mean number of deliveries per cluster per month; table 1).

**Table 1:** Characteristics of all clusters, including month-by-month variation throughout the course of the study

			**Pre-intervention deliveries (n=247 238)**	**Post-intervention deliveries (n=288 995)**	**Mean throughout trial**	**Percentage rate of change per month (95% CI)**	**p value**	**Percentage difference (95% CI)**	**p value**
Number of deliveries per month			2472 (1569)	2890 (2291)	2681 (1970)	3% (−5 to 11)	0·46	418% (−713 to 1567)	0·49
Place of delivery									
	Central referral facility		1434 (63·4%)	1282 (55·0%)	1358 (59·2%)	0·1% (−0·3 to 0·4)	0·79	−8·4% (−17·6 to 0·8)	0·11
	Peripheral facility		958 (32·8%)	1523 (41·6%)	1241 (37·2%)	0·1% (−0·2 to 0·4)	0·68	8·7% (−0·4 to 17·9)	0·093
	Home		105 (4·8%)	118 (4·7%)	111 (4·8%)	−0·1% (0·3 to 0·1)	0·28	−0·1% (−2·4 to 2·2)	0·92
Mode of delivery									
	Caesarean section		366 (16·8%)	494 (18·1%)	430 (17·4%)	0·3% (−0·1 to 0·6)	0·19	1·3% (−2·4 to 5·0)	0·52
Total number of facilities*									
	Number of primary level care facilities		232	224	228	0 (no trend)	..	−1·0% (−8·0 to 7·0)	0·85
	Number of secondary level care facilities		44	36	40	0 (no trend)	..	1·0% (−2·0 to 1·0)	0·44
	Number of tertiary level care facilities		10	10	10	0 (no trend)	..	0 (no trend)	..
Total number of facilities (per 1000 deliveries)									
	Number of primary level care facilities		13·8 (15·3)	14·3 (15·7)	14·1 (15·5)	0·0% (−0·4 to 0·4)	0·96	0·4% (−3·9 to 4·7)	0·85
	Number of secondary level care facilities		2·8 (5·4)	2·5 (5·5)	2·6 (5·5)	0·0% (−0·1 to 0·1)	0·97	−0·3% (−1·9 to 1·2)	0·66
	Number of tertiary level care facilities		0·8 (1·4)	0·8 (1·2)	0·8 (1·3)	−0·0%† (−0·0 to 0·0)	0·74	0·0% (−0·3 to 0·4)	0·89
Obstetric resources									
	Capacity for blood transfusion, mean percentage of facilities (SD)		28·9% (21·7)	21·3% (16·4)	25·1% (19·5)	−0·1% (−0·3 to 0·1)	0·42	−7·7% (−22·4 to 7·1)	0·34
	Adult intensive care unit beds‡		11·7 (9·1)	10·1 (9·1)	10·9 (9·1)	−0·1% (−0·2 to 0·1)	0·35	−1·6% (−6·0 to 2·8)	0·49
	Magnesium sulphate available, mean percentage of facilities (SD)		76·2% (25·2)	73·1% (23·7)	74·7% (24·5)	0·2% (−0·1 to 0·6)	0·21	−3·0% (−16·2 to 10·1)	0·66
Personnel (per 1000 deliveries)									
	Total doctors in maternity units		40·0 (33·4)	38·4 (28·7)	39·2 (31·1)	0·0% (−0·3 to 0·4)	0·86	−1·6% (−13·7 to 10·5)	0·80
		Obstetricians or gynaecologists	8·9 (8·3)	9·3 (7·6)	9·1 (8·0)	0·0% (−0·1 to 0·1)	0·82	0·4% (−2·4 to 3·3)	0·78
		Clinical officers	30·5 (34·3)	20·8 (23·0)	25·6 (29·5)	−0·0%† (−0·2 to 0·1)	0·61	−9·5% (−26·6 to 7·4)	0·30
		Anaesthetists (doctors)	10·0 (11·1)	6·9 (9.8)	8·5 (10·5)	0·1% (−0·0 to 0·1)	0·17	−3·1% (−9·5 to 3·2)	0·36
		Staff members trained as anaesthetists available 24 h	4·0 (3·3)	5·7 (4·7)	4·9 (4·2)	−0·0%† (−0·1 to 0·0)	0·42	1·7% (−1·4 to 4·9)	0·31
		Midwives	67·2 (57·4)	53·7 (41·6)	60·4 (50·5)	0·3% (0·03 to 0·6)	0·056	−13·5% (−48·8 to 21·7)	0·47
		Nurses with midwifery training	77·0 (45·9)	104·6 (86·2)	90·8 (70·3)	−0·5% (−1·8 to 0·9)	0·51	27·6% (−29.7 to 85·0)	0·37

Data are mean (SD) or n (%) unless otherwise stated.

* Primary level care is defined as the first point of access (eg, district clinic, clinic, or rural health post). Secondary level care is defined as a first referral point (eg, regional hospital). Tertiary level care is defined as the specialty referral centre (eg, national hospital).

† Negative values between 0·00 and −0·05.

‡ Adult intensive care unit is defined as a separate ward or room offering a higher level of care than the main ward.

Between April 1, 2016, and Nov 30, 2017, 4067 women had a primary outcome (one or more of maternal death [n=998], eclampsia [n=2692], and hysterectomy [n=681]) from 536 223 deliveries (table 2). There was an 8% reduction in the primary outcome in the intervention period compared with the pre-intervention period (79·4 per 10 000 deliveries pre-intervention to 72·8 per 10 000 deliveries post-intervention; OR 0·92, 95% CI 0·86–0·97; p=0·0056; table 2). However, after prespecified adjustments (primary analysis) for variation between and within clusters over time, no significant benefit or harm could be attributed to the intervention (OR 1·22, 95% CI 0·73–2·06; p=0·45; table 2). The calculated intracluster correlation coefficient was 0·61, much higher than the assumed 0·0085.

**Table 2:** Primary outcome, and secondary maternal and perinatal outcomes

	**Pre-intervention deliveries (n=247 238)**		**Post-intervention deliveries (n=288 995)**		**Unadjusted comparison (step)**		Planned adjusted comparison (trend and step)*		Adjusted comparison (bent stick)*	
	n (%) or n/N (%)	Rate	n (%) or n/N (%)	Rate	Odds ratio (95% CI)	p value	Odds ratio (95% CI)	p value	Odds ratio (95% CI)	p value
**Primary outcome**										
Composite (one or more of eclampsia, hysterectomy, or maternal death)	1963 (0·8%)	79·4 per 10 000 deliveries	2104 (0·7%)	72·8 per 10 000 deliveries	0·92 (0·86–0·97)	0·0056	1·13 (0·85–1·51)	0·40	1·22 (0·73–2·06)	0·45
**Secondary maternal outcomes**										
Eclampsia	1314 (0·5%)	53·1 per 10 000 deliveries	1378 (0·5%)	47·7 per 10 000 deliveries	0·90 (0·83–0·97)	0·0048	1·30 (0·82–2·05)	0·27	1·91 (0·91–4·03)	0·088
Hysterectomy	316 (0·1%)	12·8 per 10 000 deliveries	365 (0·1%)	12·6 per 10 000 deliveries	0·99 (0·85–1·15)	0·88	0·87 (0·50–1·52)	0·63	0·21 (0·07–0·66)	0·0072
Maternal death	451 (0·2%)	18·2 per 10 000 deliveries	547 (0·2%)	18·9 per 10 000 deliveries	1·04 (0·92–1·18)	0·56	0·85 (0·65–01·10)	0·22	0·80 (0·30–2·09)	0·64
Stroke	13 (<0·1%)	0·5 per 10 000 deliveries	9 (<0·1%)	0·3 per 10 000 deliveries	..	..	..	..	..	..
Admission to an intensive care unit	365 (0·1%)	14·8 per 10 000 deliveries	232 (0·1%)	8·0 per 10 000 deliveries	..	..	0·60 (0·39–0·91)	..	0·79 (0·53–1·17)	..
**Secondary perinatal outcomes**†										
Stillbirth	343/1782 (19·2%)	192 per 1000 pregnancies	500/1933 (25·9%)	259 per 1000 pregnancies	..	..	1·02 (0·61–1·69)	..	0·95 (0·87–1·04)	..
Neonatal death	52/1782 (2·9%)	29 per 1000 pregnancies	77/1933 (4·0%)	40 per 1000 pregnancies	..	..	..	..	..	..

* Adjusted for cluster effect, time from start of study (with an interaction between cluster and time so that each cluster had its own underlying time trend), and total time on the randomised intervention.

† In women with a primary outcome with delivery information. Excludes 17 women with missing delivery information, 45 women who went home after a primary outcome without delivery and were not followed up, and 290 women who were less than 28 weeks pregnant at the time of the primary outcome and delivery data were not collected.

Analysis was undertaken using alternative correlation structures as planned, with no significant findings (appendix p 6). Prespecified sensitivity analysis removing four periods of data during which there were external changes within the site (strike action affecting staffing levels in three sites and natural disaster in Haiti) had no impact on the results.

A prespecified analysis of the individual components of the primary outcome found a significant reduction in the rate of emergency hysterectomy in the intervention period compared with the pre-intervention period (table 2), but the eclampsia and maternal death rates did not significantly change (table 2). Very few women had a stroke, with 13 in the pre-intervention period and nine in the intervention period (convergence for comparison not achieved). There was no significant change in the number of women admitted to intensive care (table 3).

**Table 3:** Additional information on primary outcomes

	**Pre-intervention**		**Post-intervention**		Planned adjusted comparison (trend and step)*	Adjusted comparison (bent stick)*
	n/N (%)	Rate per 10 000 deliveries	n/N (%)	Rate per 10 000 deliveries		
**Place of death (as % of all deaths)**						
Central referral facility	428/451 (94·9%)	..	522/546 (95·6%)	..	..	..
Peripheral facility	12/451 (2·7%)	..	17/546 (3·1%)	..	..	..
Community	11/451 (2·4%)	..	7/546 (1·3%)	..	..	..
**Cause of death**						
Obstetric haemorrhage	147/451 (32·6%)	6·0	212/546 (38·8%)	7·3	0·86 (0·56–1·33)	0·56 (0·29–1·05)
Pregnancy-related sepsis	67/451 (14·9%)	2·7	74/546 (13·6%)	2·6	..	..
Other sepsis	15/451 (3·3%)	0·6	13/546 (2·4%)	0·5	..	..
Hypertensive disorder in pregnancy (eclampsia, pre-eclampsia, or stroke)	81/451 (18·0%)	3·3	123/546 (22·5%)	4·3	0·76 (0·46–1·25)	2·07 (0·33–13·12)
Other	141/451 (31·3%)	5·7	125/546 (22·9%)	4·3	0·88 (0·62–1·24)	0·54 (0·05–5·73)
**Place of first eclamptic fit**						
Central referral facility	506/1314 (38·5%)	20·5	333/1378 (24·2%)	11·5	0·56 (0·33–0·97)	1·17 (0·53–2·55)
Peripheral facility	280/1314 (21·3%)	11·3	363/1378 (26·3%)	12·6	1·55 (1·10–2·20)	1·35 (0·17–10·45)
Community	528/1314 (40·2%)	21·4	682/1378 (49·5%)	23·6	1·02 (0·56–1·86)	2·78 (0·65–11·89)
**Cause of hysterectomy**						
Postpartum haemorrhage	112/316 (35·4%)	4·5	148/365 (40·5%)	5·1	1·23 (0·72–2·10)	0·45 (0·11–1·09)
Ruptured uterus	151/316 (47·8%)	6·1	172/365 (47·1%)	6·0	0·87 (0·41–1·84)	0·12 (0·02–0·82)
Sepsis	21/316 (6·6%)	0·8	25/365 (6·8%)	0·9	..	..
Other	32/316 (10·1%)	1·3	20/365 (5·5%)	0·7	..	..

Data are n/N (%) or odds ratio (95% CI) unless otherwise stated.

* Adjusted for cluster effect, time from start of study (with an interaction between cluster and time so that each cluster had its own underlying time trend), and total time on the randomised intervention.

In delivery data available for 3715 women with a primary outcome (including 123 twin and two triplet pregnancies), there were 843 (22·7%) stillbirths, with no significant difference between groups (table 2).

Nearly all maternal deaths (950 [95·2%] of 997) occurred in central referral facilities (table 3). There were no significant changes in the cause of maternal death between groups (table 3). The highest proportion of first eclamptic fits occurred in the community. After adjustments, there were no significant changes between groups in the place of first fit. 323 (47·4%) of 681 emergency hysterectomies were performed for ruptured uterus, with 260 (38·2%) for postpartum haemorrhage alone. After adjustments, there were no significant changes in the cause of hysterectomy between groups (table 3).

**Figure 3:**
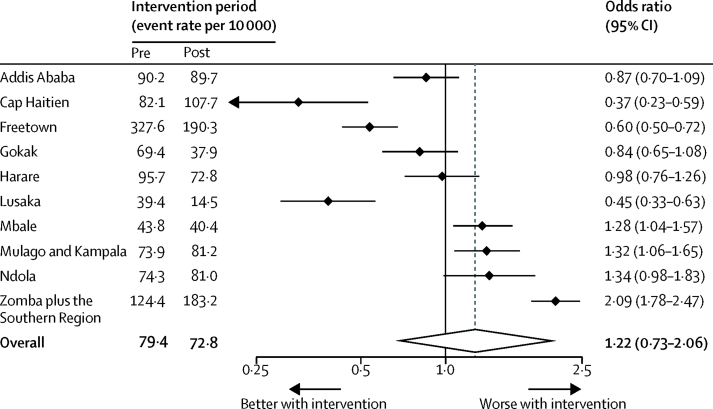
Forest plot of event rates in individual clusters and the effect of the intervention on the primary composite outcome analysed with fixed linear trends

Individual site analysis is limited because clusters were purposely selected and these data are non-randomised. However, individual analysis is presented as planned in figure 3. The event rate in the pre-intervention period ranged from 39·4 per 10 000 deliveries in Lusaka to 327·6 per 10 000 deliveries in Freetown. After adjustment, there was considerable heterogeneity in the apparent effect in individual clusters (*I*^2^=94·5%), with significant benefit shown in three sites, including the two clusters with the highest and lowest baseline event rate.

Training was delivered to 2747 health-care providers, with 3868 devices distributed across 286 facilities. On average, 2784 (3·7%) of 74 828 women seen in peripheral maternity facilities were referred in the pre-intervention period compared with 3212 (4·4%) of 73 371 women in the intervention period (OR 0·89, 95% CI 0·39–2·05; data from the Mulago cluster are excluded because they were not able to collect the denominator). However, changes in referral varied between sites, with a significant reduction in referral rates in five sites, no change in three sites, and a 16-fold increase in referrals in a single site (Gokak). This increase is thought to be due to the high incidence of severe anaemia in Gokak resulting in frequent referrals of asymptomatic women due to an elevated shock index (this is under further investigation). Overall, there were no changes in staffing or resource levels. By the end of the trial, 0·6% (n=23) of CRADLE VSAs were reported missing and 4·6% (n=180) reported to be broken. The most commonly reported reasons were failure of the battery, leaking of the valve in the pump, or tears in the cuff.

## Discussion

This trial was unable to demonstrate a direct effect of the CRADLE intervention on a composite outcome of maternal mortality and morbidity, although together these outcomes fell by 8% during the trial. There was considerable heterogeneity of data between and within sites, giving insufficient power despite more than 4000 primary events, the number of which was higher than anticipated. Prespecified secondary analyses showed no significant reduction in maternal death or eclampsia but a significant reduction in emergency hysterectomy. Further work is required to elucidate the importance of this result. It is plausible that this reduction represents a true benefit, potentially as a result of earlier referral for post-partum haemorrhage, as we hypothesised.

The strengths of this study included the randomised design in multiple countries, size of the trial population, use of routine data triangulated with active case finding, implementation into all levels of health-care facilities within clusters, and eligibility of all pregnant women. A limitation was that implementation and data collection were by the same team, introducing possible measurement bias. It is also plausible that in some clusters, use of the intervention might have resulted in increased reporting of the primary outcome if previously occurring without documentation in the community, with a bias against the intervention. However, case finding and data collection were carefully optimised in the feasibility phase and closely monitored by the local investigator and research team.

The stepped-wedge randomised controlled trial design was chosen because phased implementation across ten clusters was more feasible than simultaneous implementation and because it would have been challenging to achieve sufficient cluster matching. In addition, because blood pressure measurement is part of routine maternity care and adequate access to equipment in low-resource settings is a challenge, delivery to all clusters was deemed preferable by our sites. However, this trial design also reflects a study limitation because it is vulnerable to temporal trends and external influences, although extensive efforts were made to capture and adjust for these factors. The decision to involve diverse clusters across eight countries was made to enable generalisability. We have demonstrated successful intervention delivery in multiple settings. Variation between clusters was taken into account in the sample size calculation and the randomisation procedure, but there were no reliable data on which to adjust for temporal and seasonal trends. These seasonal variations were larger than anticipated and, together with the size and complexity of the temporal trends, had a substantial impact on power, despite the large total number of events.

Although stepped-wedge randomised controlled trials are becoming increasingly popular,^32^, ^33^ this trial was unusual in being undertaken across multiple countries and presents valuable learning for others planning multi-country stepped-wedge randomised controlled trials. We can only identify one published trial undertaken in multiple countries (in Europe).^34^ Compared with parallel-cluster trial designs, stepped-wedge randomised controlled trials remain an appropriate solution with an intracluster correlation coefficient anticipated to be up to 0·1,^28^ but the potential variation between clusters (and logistical challenges) are magnified when the study involves multiple countries.

Across all our sites, there was an 8% reduction in the primary outcome during the trial period. The statistical analysis accounts for event rate trends in each cluster before and after the intervention. After adjusting for these trends, this reduction cannot be directly attributed to the intervention. Trends from WHO indicate that over the past 5 years, the mean reduction in maternal mortality ratio across the eight countries in this trial was only 2% per year (range −1% in Malawi to 22% in Ethiopia).^1^ There is a scarcity of reliable prevalence trends for eclampsia and maternal morbidity in these countries because data are primarily limited to intervention studies or small observational studies. It is plausible that the intervention was beneficial but not proven by our trial design or that participation in the trial and the process of data collection were associated with benefit.^35^

Behavioural change theory states that to be effective, interventions should target specific behaviours and that success is dependent on having the necessary skill and intention, in the absence of environmental constraints.^36^ The CRADLE intervention incorporated the device with an educational package and clinical champions to promote the importance of vital signs measurement in pregnancy. We hypothesised that this intervention would improve the number of women that receive vital signs monitoring and subsequent management of pregnancy complications. Following identification of complications, prevention of morbidity and mortality is dependent on capacity of health-care providers and the health-care system to respond. Although desirable, it was not feasible to measure individual abnormal vital signs and clinical management. Accurate measures of quality of care are challenging in low-resource settings and can be associated with methodological issues;^37^, ^38^ therefore, it is a strength that important, unequivocal clinical outcomes were measured. A mixed-methods process evaluation was undertaken in parallel to this trial, which measured implementation (fidelity, dose, and reach) and explored potential mechanisms of the intervention in each cluster. Further analysis of these results, exploring whether these measures, in combination with staffing and resource levels, affect the effect of the intervention in different clusters, will be published.

In conclusion, the CRADLE intervention was successfully implemented into routine maternity care in low-resource settings. The intervention was associated with a reduction in a composite of death, eclampsia, and hysterectomy but only hysterectomy was directly attributed to the intervention. The trial had insufficient power because of unexpected variation between clusters and despite the high number of primary outcome events. Potential variation between and within clusters, over time, should be taken into consideration in planning future stepped-wedge randomised controlled trials. Future research could consider a cluster or stepped-wedge randomised controlled trial in a single country that should use extensive pilot data (including accurate intracluster correlation and baseline temporal trends) to inform the power calculation. In-depth analysis of clinical care pathways in a subset of districts could be considered. Effects on individual sites and components of the primary outcome in relation to availability of resources and staffing need investigation.

## Data sharing

The dataset will be available to appropriate academic parties on request from the Chief Investigator in accordance with the data sharing policies of King's College London (UK), with input from the Co-investigator group where applicable.
