# Supplementary material for: Effect of a novel vital sign device on maternal mortality and morbidity in low-resource settings: a pragmatic, stepped-wedge, cluster-randomised controlled trial
Source: Lancet Glob Health. 2019 Feb 14;7(3):e347–56. doi: 10.1016/S2214-109X(18)30526-6 (PMC6379820; doi:10.1016/S2214-109X(18)30526-6)
Supplement: Supplementary appendix [file mmc1.pdf]

THE LANCET
Global Health

Supplementary appendix

This appendix formed part of the original submission and has been peer reviewed. We post it as supplied by the authors.

Supplement to: Vousden N, Lawley E, Nathan HL, et al. Effect of a novel vital sign device on maternal mortality and morbidity in low-resource settings: a pragmatic, stepped-wedge, cluster-randomised controlled trial. *Lancet Glob Health* 2019; **7:** e347–56.

Appendix Content

1. **Ethical approval for clusters** **Page 2**
2. **List of facilities in each cluster** **Page 3-5**
3. **Alternate correlation models** **Page 6**
4. **Affiliations of collaborators** **Page 7-8**

1. **Ethical Approval for Sites**

Country	Cluster Primary Investigator and Affiliation	Ethics Approval Authority	Ethics Approval Number
Ethiopia	Adrian Brown, Maternity Worldwide.	Ethiopian Public Health Institute, Ethiopia	EPHI6.4/185
Zimbabwe	Francis Gidiri, University of Zimbabwe.	Medical Research Council of Zimbabwe; Zimbabwe.	MRCZ/A/1999
Sierra Leone	Matthew Clarke, Welbodi Partnership, Freetown.	Office of the Sierra Leone Ethics and Scientific Review Committee Directorate of Training and Research, Connaught Hospital; Sierra Leone	Not provided
Haiti	Carwyn Hill, Hope Health Action, Cap Haitien	Cap Haitien does not have a formal ethical review process, Memorandums of understanding were drawn up with each hospital trust and a letter of support gained from the Ministry of Health.	NA
India	Mrutyunjaya Bellad, Jawarharlal Nehru Medical College, KLE University, Belgaum.	K.L.E Society's Jawaharlal Nehru Medical College, Belgaum, India	MDC/IECHSR/2015-16/A-59; KLEU/EC/2016-17/A-95; KLEU/EC/2017-18-A-104
Zambia	Bellington Vwalika, University of Zambia, Lusaka; Sebastian Chinkoyo, Ndola Teaching Hopital, Ndola.	ERES Converge; Zambia	20215-Aug-008
Malawi	Grace Makonyola, Maternity Worldwide.	National Health Sciences Research Committee at Zomba Central Hospital, Malawi	NHSRC 15/11/1504
Uganda	Julius Wandabwa, Sanyu Africa Research Institute, Mbale; Josephat Byamugisha, Makere University,	Uganda National Council for Science and Technology; Uganda	HS1953

2. Trial Facilities in each cluster

Cluster	Tertiary Facilities	Secondary Facilities	Primary Facilities
Addis Ababa, Ethiopia	St Paul's Hospital, Addis Ababa, Ethiopia	Ras Desta, Addis Ababa, Ethiopia	Selam Health Center (Woreda 9), Woreda 7 Health Centre (Gulele)/Hidasse, Woreda 10 Health Center (Gulele)/shegole, Free Methodist Health Center, Woreda 5 Health Center, Woreda 2 Health Center Michewe, Addis Ketema Health Center (Addis Ababa), Woreda 5/18 Health Center (Addis Ketema), Woreda 7 Health Center (Addis Ketema), Woreda 7 Health Center (Addis Ketema), Woreda 10 Addis Ketema, Woreda 5 Abebe Bikila, Ras Emiru Health center, Gulele Semen, Simgn Kebede, Kolfe Woreda 2, Addis Gebeya HC, Mikililand Health Centre
Harare, Zimbabwe	Mbuya Nehanda Maternity Hospital, Harare, Zimbabwe	Concession District Hospital<br>Makumbi Hospital	Henderson Clinic, Christon Bank Clinic, Nyabira Clinic, Gwebi College Cllnic, Mount Hampden Clinic, Dzivarasekwa Extension Clinic, St Josephs Clinic, Mabvuku Polyclinic, Warren Park Polyclinic, Hatcliff Polyclinic, Rujeko (DZ) Polyclinic, Parirenyatwa City clinic, Tafara Clinic, Greendale Clinic, Eastlea Clinic, Highlands Clinic, Borrowdale Clinic, Mt Pleasant Clinic, Avondale Clinic, Belvedere Clinic, Mabelreign Clinic, Malborough Clinic
Ndola, Zambia	Ndola Teaching Hospital	N/A	Chipokota Mayamba Clinic, Chipulukusu Clinic, Commando Camp Clinic, Dola Hill Clinic, Itawa Clinic, Kabushi Clinic, Kalewa Clinic, Kaloko Clinic, Kaniki Clinic, Kawama Clinic, Lubuto Clinic, Main Masala Clinic, Mushili Clinic, Ndeke Clinic, New Masala Clinic, Nkwazi Clinic, Padmodzi Clinic, Peter Singogo Clinic, Prisons Clinic, Railway Surgery, St Dominic Mission Hospital, Tug Argan Clinic, Twapia Clinic
Freetown, Sierra Leone	Princess Christian Maternity Hospital, Freetown, Sierra Leone	Rokupa Government Hospital	Approve School CHC, Haja Neneh, Jenner Wright CHC, Kissy CHC, Konkay CHC, Kuntorloh CHC, Looking Town MCHP, Moyiba CHC, Principal Medical officer Clinic, Ross Road CHC, St. Joseph CHC
Cap Haitien, Haiti	Fort Saint Michel Hopital, Convention Baptiste d'Haiti,	Centre de Sante Quartier-Morin	Centre de Sante de Cadush, Centre de Sante de Morne Pele, Centre de Sante Labadie, Centre de Sante Limonade, Centre de Sante Porte Ouverte, Centre de Sante St Charles, Dispansaire St. Louis, Dispensaire de Grand Pre, Unite

	Justinian University Hospital		de Lutte pour la Sante (ULS), Centre de Sante de Madeline
Gokak, India		Al Shifa Hospital<br>Arogya hospital Mudalgi<br>Arogya woman child Hospital,<br>Mahila and Childrens Hospital<br>Dhondiba Jadhav Memorial Hospital,<br>Dr Kattimani Hospital<br>Ganga surgical and Maternity Clinic<br>Gokak General Hospital<br>Gourishankar Hospital Gokak<br>J G Cooperative Hospital<br>Jayaratna Hospital,<br>Kadagalikar Maternity and Children Hospital,<br>Kappalaguddi Hospital,<br>KHI Hospital<br>Masurkar Hospital,<br>Mudalagi CHC<br>Muragod Hospital,<br>Navajeevan Maternity & Nursing Home,<br>Nayakwadi Hospital,<br>Shanta Nursing and Maternity Home<br>Soubhagya Nursing and Maternity Home	Akkatangerhal SC Dasanatti, Akkatangerhal (PHC+ Sub Centres AK Hal I & II, Akkatangerhal SC Iranatti, Akkatangerhal SC Panjanatti, Ankalagi (PHC+ Sub Centres I and II), Ankalagi SC Gujanal, Ankalagi SC Mallapur, Ankalagi SC Suladal, Bairanatti (PHC+ Sub Centres I), Bairanatti SC Sunadholi, Bairanatti SC Tigadi, Balobal SC Hunshyal, Balobal SC Sangankeri, Balobal (PHC and SC I), Balobal SC Arabhavi, Balobal SC Lolasur, Betageri SC Chikkanandi, Betageri (PHC and SC I), Company Hospital Gokak Falls, Hallur (PHC and SCI and II), Hallur SC Khanatti, Kallolli (PHC and SC I, II), Khanagaon (PHC and SCI), Khanagaon SC DG Hatti, Khanagaon SC Shiltibhavi, Konnur (PHC SC I and II), Konnur SC Godachinamalki, Konnur SC Gokak Falls i and II, Konnur SC Melamatti, Konnur SC Nandagaon, Koujalagi (PHC and SC I and II), Koujalagi SC Kalliguddi, Kulagod (PHC SC I and II), Kulagod SC Dhavaleshwar, Mamadapur (PHC and SCI), Mamadapur SC Maradishivapur, Masaguppi (PHC SCI), Masaguppi SC Dharmatti, Masaguppi SC Vadratti, Melavanki PHC (PHC+ SC-1), Melavanki PHC -SC Maladinni, Melavanki PHC -SC Upparatti, Naganur (PHC SCI), Naganur SC Gurlapur, Naganur SC Mudalagi I and II, Sindhikurabet (PHC SC I and II), Sindhikurabet Ghataprabha I and II, Sindhikurabet SC Dupdhal, Sindhikurabet SC Duradundi, Talakatnal (PHC and SC), Talakatnal SC Gosabal, Talakatnal SC Uddagatti, Tavag (PHC SC I and II), Tavag SC Benachinamardi, Tavag SC Kolavi, Tavag SC Urabinatti, Tukkanatti (PHC SC), Tukkanatti SC PG Mallapur, Tukkanatti SC Rajapur, Yadawad (PHC and SC), Yadawad SC Avaradi, Yadawad SC Girisagar, Yadawad SC Yaragudri
Mulago, Uganda	Mulago and Kawempe Hospital	Lubaga Hospital,<br>Mengo Hospital,<br>Nsambya Hospital	Kawala Health Centre III, Kisenyi Health Centre, Kisugu Health Centre III, Kiswa Heaslth Centre III, Kitebi Health Centre III, Komamboga Health Centre III, Naguru general hospital, Naguru Teenage Centre, Kibuli Hospital
Lusaka, Zambia	University Teaching Hospital	Chainama, Chainda, Chawama, Kalingalinga, Kanyama,	Bauleni, Chaisa, Chazanga, Chelstone, Civic Center, George, Kabwata, Kamwala, Kaunda Square, Matero

		Mtendere, Chilenje, Chipata, Levy Hospital, Matero Referral, Sikanze	Main, Ngombe, Prisons, Railway, State House, State Lodge
Zomba, Malawi	Zomba central Hospital	Balaka , Holy Family, Machinga, Mangochi, St Lukes, Pirimiti	Chingale, Chipini, Magomero, Matawale, Matiya, Mayaka Namikango , Ntaja, Phalombe
Mbale, Uganda		Mbale regional referral hospital	Ahamadiya. Atuturi hospital, Bubulo, Budadiri, Budaka<br>Bududa hospital, Bufumbo<br>Bugobero, Bukedea, Bukiende, Bumadanda, Bumasike, Bunampogo, Bungokho, Busano, Bushikori, Busiu, Busolwe Hospital, Buwangwa, Kadama, Kamonkoli, Kibuku, Kolonyi, Lwangoli, Makhonje, Maluku, Mbale prisons, Mt.elgon hospital, Naiku, Nakaloke, Namakwekwe, Namanyonyi, Namatala, Namawanga, Pallisa hospital, Police 2, Siira HCIII, Sironko, Tirinyi, Wanale

3. Alternative Correlation Models Tested

Correlation Model	Planned Adjusted Comparison* (Trend & Step) (Odds Ratio)	Adjusted Comparison* (Bent Stick) (Odds Ratio)
Ar 1	1.12 (0.86-1.47)<br>0.39	1.11 (0.80-1.54)<br>0.54
Ar 5	1.13 (0.85-1.51)<br>0.40	1.17 (0.70-1.95)<br>0.70
Exchangeable	No convergence	No convergence
Independent	1.32 (0.86-1.48)<br>0.37	1.13 (0.81-1.57)<br>0.81
Unstructured	No convergence	No convergence
Stationary 1	1.13 (0.86-1.47)<br>0.39	1.11 (0.80-1.55)<br>0.53
Stationary 5	1.11 (0.84-1.45)<br>0.48	No convergence
Non-stationary 1	No convergence	No convergence
Non-stationary 5	No convergence	No convergence

4. Affilliations of Collaborators

First name	Last name	Affiliation
Monice	Kachinjika	Maternity Worldwide, Zomba, Malawi
Doreen	Bukani	Maternity Worldwide, Zomba, Malawi
Jane	Makwakwa	Maternity Worldwide, Zomba, Malawi
Grace	Makonyola	Maternity Worldwide, Zomba, Malawi
Adrian	Brown	Maternity Worldwide, Community Base, 113 Queens Rd, Brighton BN1 3XG
Paul	Toussaint	Hope Health Action, Hopital Convention Baptiste d'Haiti, Cap Haitien, Haiti
Adeline	Vixama	Hope Health Action, Hopital Convention Baptiste d'Haiti, Cap Haitien, Haiti
Grace	Greene	Hope Health Action, Hopital Convention Baptiste d'Haiti, Cap Haitien, Haiti
Carwyn	Hill	Hope Health Action, Hopital Convention Baptiste d'Haiti, Cap Haitien, Haiti
Emily	Nakiriija	Department of Obstetrics and Gynaecology, Mulago Hospital, Makerere University, Kampala, Uganda
Doreen	Birungi	Department of Obstetrics and Gynaecology, Mulago Hospital, Makerere University, Kampala, Uganda
Noela	Kalyowa	Department of Obstetrics and Gynaecology, Mulago Hospital, Makerere University, Kampala, Uganda
Dorothy	Namakula	Department of Obstetrics and Gynaecology, Mulago Hospital, Makerere University, Kampala, Uganda
Josaphat	Byamugisha	Department of Obstetrics and Gynaecology, Mulago Hospital, Makerere University, Kampala, Uganda
Annettee	Nakimuli	Department of Obstetrics and Gynaecology, Mulago Hospital, Makerere University, Kampala, Uganda
Nathan	Mackayi Odeke	Sanyu Africa Research Institute, Mbale Regional Referral Hospital, Mbale, Uganda
James	Ditai	Sanyu Africa Research Institute, Mbale Regional Referral Hospital, Mbale, Uganda
Julius	Wandabwa	Sanyu Africa Research Institute, Mbale Regional Referral Hospital, Mbale, Uganda
Fatmata	Momodou	Welbodi Partnership, Ola During Childrens Hospital, Freetown, Sierra Leone
Margaret	Sesay	Welbodi Partnership, Ola During Childrens Hospital, Freetown, Sierra Leone
Patricia	Sandi	Welbodi Partnership, Ola During Childrens Hospital, Freetown, Sierra Leone
Jeneba	Conteh	Welbodi Partnership, Ola During Childrens Hospital, Freetown, Sierra Leone
Jesse	Kamara	Welbodi Partnership, Ola During Childrens Hospital, Freetown, Sierra Leone
Matthew	Clarke	Welbodi Partnership, Ola During Childrens Hospital, Freetown, Sierra Leone
Rebecca	Best	Welbodi Partnership, Ola During Childrens Hospital, Freetown, Sierra Leone
Josephine	Miti	Department of Obstetrics and Gynaecology, University of Zambia, Lusaka, Zambia
Mercy	Kopeka	Department of Obstetrics and Gynaecology, University of Zambia, Lusaka, Zambia
Bellington	Vwalika	Department of Obstetrics and Gynaecology, University of Zambia, Lusaka, Zambia
Martina	Chima	Department of Obstetrics and Gynaecology, University of Zambia, Lusaka, Zambia
Thokozile	Musonda	Department of Obstetrics and Gynaecology, Ndola Teaching Hospital, Ndola, Zambia

Sebastian	Chinkoyo	Department of Obstetrics and Gynaecology, Ndola Teaching Hospital, Ndola, Zambia
Christine	Jere	Department of Obstetrics and Gynaecology, Ndola Teaching Hospital, Ndola, Zambia
Violet	Mambo	Department of Obstetrics and Gynaecology, College of Health Sciences, University of Zimbabwe, Zimbabwe
Yonas	Guchale	Maternity Worldwide, Addis Ababa, Ethiopia
Lomi	Yadeta	Maternity Worldwide, Addis Ababa, Ethiopia
Feiruz	Surur	Department of Obstetrics and Gynaecology, St. Pauls Hospital, Addis Ababa, Ethiopia
Geetanjali M	Mungarwadi	Women's and Children's Health Research Unit, KLE Academy of Higher Education and Research, Jawaharlal Nehru Medical College, Belgaum – 590010 Karnataka, India
Sphoorthi S	Mastiholi	Women's and Children's Health Research Unit, KLE Academy of Higher Education and Research, Jawaharlal Nehru Medical College, Belgaum – 590010 Karnataka, India
Chandrappa C	Karadiguddi	Women's and Children's Health Research Unit, KLE Academy of Higher Education and Research, Jawaharlal Nehru Medical College, Belgaum – 590010 Karnataka, India
Umesh	Charantimath	Women's and Children's Health Research Unit, KLE Academy of Higher Education and Research, Jawaharlal Nehru Medical College, Belgaum – 590010 Karnataka, India
Mrutyunjaya	Bellad	Women's and Children's Health Research Unit, KLE Academy of Higher Education and Research, Jawaharlal Nehru Medical College, Belgaum – 590010 Karnataka, India
Natasha	Hezelgrave	Department of Women and Children's Health, School of Life Course Sciences, Faculty of Life Sciences and Medicine, King's College London, London, SE1 7EH
Kate E	Duhig	Department of Women and Children's Health, School of Life Course Sciences, Faculty of Life Sciences and Medicine, King's College London, London, SE1 7EH
